# Intravascular Lithotripsy for Underexpanded Stent in Heavily Calcified Coronary Artery Disease

**DOI:** 10.1155/2022/7075933

**Published:** 2022-07-15

**Authors:** Tejas Raxwal, Cauvery Balhara, Dipak Parekh

**Affiliations:** ^1^Access Health Care Physicians, Hudson, Florida, USA; ^2^Regional Medical Center Bayonet Point, Hudson, Florida, USA

## Abstract

**Objective:**

Can intravascular lithotripsy safely modify calcium in an underexpanded stent and result in optimal expansion and improved outcome? Coronary artery calcification results in difficulty with stent delivery and expansion and is associated with adverse outcomes. Intravascular lithotripsy (IVL) by delivering acoustic pressure waves modifies calcium plaque that improve vessel compliance resulting in optimizing stent deployment.

**Results:**

A 63-year-old patient presented with acute coronary syndrome with symptoms of unstable angina who underwent angiography that showed calcified right coronary artery which was treated with balloon angioplasty and stenting with drug eluting stent. However, after multiple inflations with noncompliant balloon, the patient was noted to have persistent residual in stent stenosis of 70%. Stenotic lesion in poorly expanded stent was treated with Shockwave C2 Coronary Intravascular Lithotripsy catheter resulting in 0% residual stenosis. The patient was followed for major adverse cardiovascular events at 30 days and 12 months. The patient remained MACE free at 30 days and 12 months.

**Conclusions:**

Coronary IVL safely and effectively facilitated stent expansion in severely calcified lesion in a poorly expanded stent with MACE free at 12-month follow-up.

## 1. Background

Severely calcified coronary arteries are known to portend lower procedural success rates and increased periprocedural complications. Lithotripsy technology is used for treatment of ureterorenal calculi, which has now been adapted as a new technique of intravascular lithotripsy (IVL) for heavily calcified lesions in coronary arteries [1].

IVL is a novel technique in which multiple lithotripsy emitters mounted on a traditional catheter platform deliver localized pulsatile sonic pressure waves to circumferentially modify vascular calcium by inducing calcium fractures. In the Disrupt CAD II study, the safety and effectiveness of IVL were reported for vessel preparation of severely calcified vessel in stenotic de novo coronary lesions before stent implantation [2].

Success and safety of the use of intravascular lithotripsy in a newly deployed underexpanded drug-eluting stent are unclear. Does it cause delamination of drug and polymer from stents and alter elution kinetics and drug delivery? Short- and long-term adverse cardiac events are unknown.

## 2. Case Report

63-year-old male has a history of diabetes, hyperlipidemia, HTN, and severe COPD and pulmonary HTN. The patient is a current smoker for over 50 years. Echocardiogram showed EF 60% with a PA pressure of 70 mmHg, and ECG showed sinus rhythm with occasional PVCs and incomplete RBBB. The patient had an episode of SVT with HR of 170 beats per minute. The patient had mildly elevated troponin of 0.15 ng/dl. Stress test showed an inferior-lateral perfusion defect in view of the fact that coronary angiography was performed.

**Figure 1:**
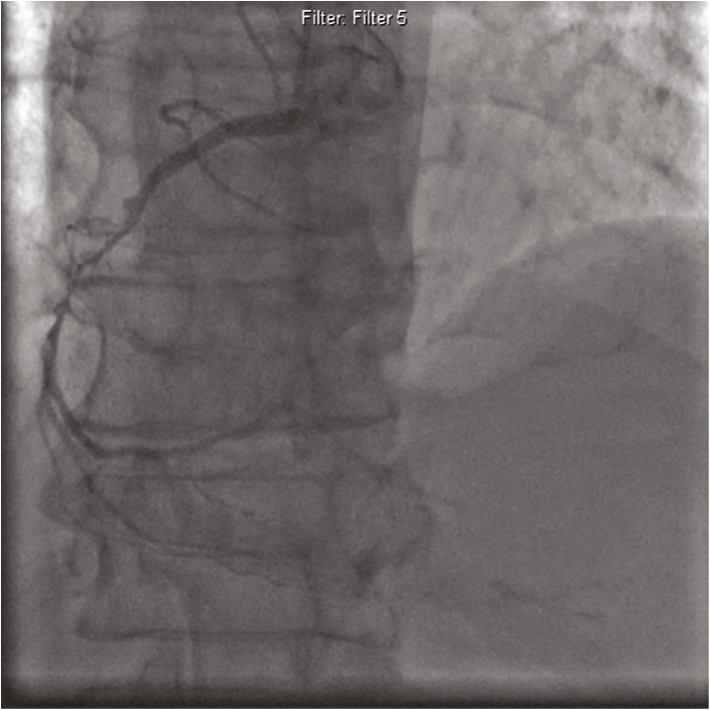
Right coronary artery with severe stenosis.

**Figure 2:**
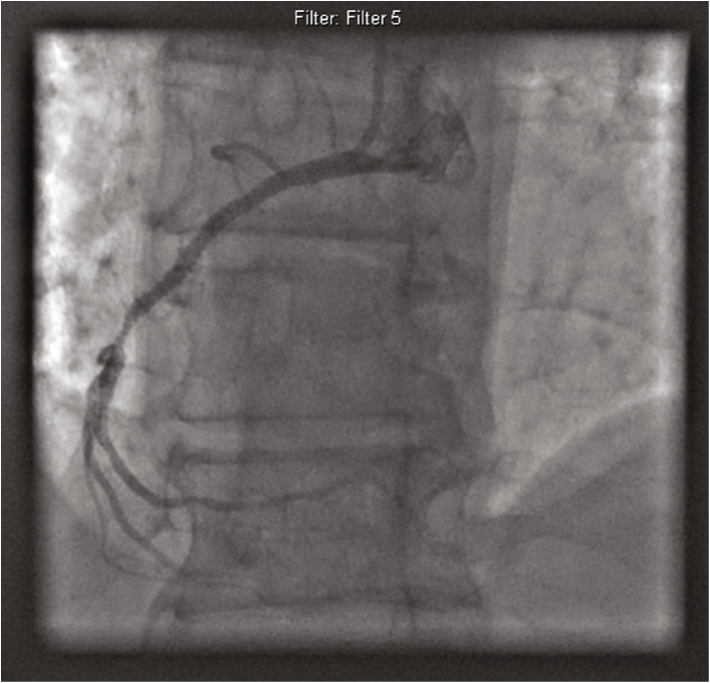
Right coronary artery with severe residual stenosis post balloon angioplasty and stenting.

Coronary angiogram showed the right coronary artery was severely diseased with 95% stenosis, so a decision was made to perform angioplasty (Figure 1). A coronary guidewire was crossed and placed in the distal PDA. A 2.5 × 12 mm compliant balloon was used to predilate the lesion. A 2.75 × 28 mm Xience DES was deployed distally, and an overlapping 3.0 × 18 mm Xience DES was deployed proximally. The stents were postdilated using a 3.5 × 12 noncompliant balloon. Repeat angiography despite multiple high pressure inflations showed the stent had a residual stenosis of approximately 70% (Figure 2). At this point, a decision was made to evaluate options of intravascular lithotripsy, laser versus coronary artery bypass surgery.

In discussion with the cardiac surgery team, it was decided attempting intravascular lithotripsy would be favorable in view of the patient having severe pulmonary HTN with severe COPD; his cardiac surgery risk would outweigh compared to attempting lithotripsy.

**Figure 3:**
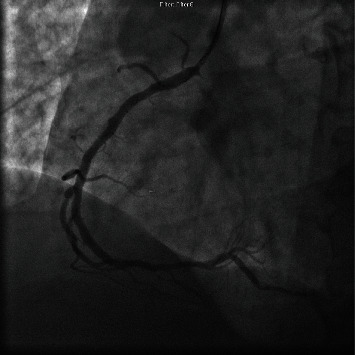
Right coronary artery after intravascular lithotripsy.

Patient was brought to the cardiac catheterization laboratory, and a Shockwave C2 Coronary Intravascular Lithotripsy catheter 3 mm × 12 mm balloon was inserted, and lithotripsy was done. Followed by lithotripsy, a 3.5 mm × 15 mm noncompliant balloon was inserted and balloon angioplasty was successfully performed which resulted in 0% residual stenosis (Figure 3).

The primary endpoint was in-hospital major adverse cardiac events (MACE) defined as cardiac death, MI, or target vessel revascularization. Secondary endpoints included angiographic success, defined as the ability of IVL to produce a residual diameter stenosis < 30% without serious angiographic complications (severe dissection impairing flow, perforation, abrupt closure, persistent slow flow, or no reflow). The patient had successful angioplasty with <10% residual stenosis facilitated by IVL with no MACE in-hospital, at 30 days and 12 months.

## 3. Discussion

Percutaneous coronary artery intervention of heavily calcified arteries remains a challenge. Specialty balloons like scoring, cutting, and ultrahigh pressure balloons have been used to pretreat these lesions. Rotational atherectomy is commonly used to facilitate PCI in calcified vessels; however, all techniques have significant limitations. Noncompliant balloon dilation, despite high pressures, may be of insufficient force to result in calcium fracture and thus artery expansion. In the presence of eccentric calcium, balloon dilation may be biased toward noncalcified segments of the artery, leading to dissection or perforation.

Rotational and orbital atherectomy, while highly effective for facilitating lesion crossing, may selectively ablate calcified segments of the artery resulting from guidewire bias, leaving significant calcium plaque unmodified within the lesion [3]. Periprocedural complications like slow-flow, periprocedural myocardial infarction, dissection, and perforation may result more often in atherectomy compared with balloon-based therapies.

Treatment of underexpanded stent still remains a challenge and poses an increased risk of early stent thrombosis and restenosis after PCI, while laser atherectomy has been used successfully in underexpanded stents using “explosion” technique [4]. However, it does have risk of dissection, perforation, and distal embolization.

In February 2021, the Shockwave C2 Coronary IVL Catheter was approved by the FDA for use before implanting a stent in heavily calcified coronary arteries. Intravascular lithotripsy is a novel technology, based on an established treatment strategy for renal calculi, delivered via a traditional catheter used to circumferentially modify vascular calcium.

The coronary IVL catheter contains multiple lithotripsy emitters enclosed in an integrated balloon. The emitters generate sonic pressure waves in the shape of a sphere, creating a field effect to treat vascular calcium. The generated sonic pressure waves selectively disrupt and fracture calcium in situ, altering vessel compliance while minimizing injury and maintaining the integrity of the fibroelastic components of the vessel wall.

Herein, we report safety and effectiveness of IVL for the modification of calcified plaque in an underexpanded stent. The IVL mechanism of action has been shown to be intraplaque calcium fracture, thereby modifying vascular compliance and facilitating stent expansion. IVL was safe with no dissection, perforation, abrupt closure, or slow flow/no reflow. There was no MACE reported at 30 days and 12 months. IVL is a feasible and safe frontline tool for calcified plaque modification, with the IVL catheter crossing the lesion and delivering therapy in complex cases including underexpanded stents.

In the Disrupt CAD study, the percentage of patients that underwent intravascular lithotripsy freedom from 30-day MACE was 92% [5, 6]. Thus, pending long-term results from the present case and additional studies, IVL may be a new, simple-to-use therapeutic option for patients with underexpanded stents as a bailout strategy to impact clinical outcomes of stent thrombosis, reduced restenosis, and reduced target lesion revascularization [7]. However, atherectomy remains the first-line therapy for optimal lesion preparation if there is difficulty in crossing the lesion with even contemporary low-profile balloon catheters.

In multicenter IVL-Dragon Registry, 62 consecutive patients with stent underexpansion were treated with IVL procedural success (72.6% of patients) [8]. Intravascular imaging confirmed increased stent expansion following IVL from 37.5% to 86% by optical coherence tomography and from 57% to 89% by intravascular ultrasound. One patient had cardiac death; however, there was no target lesion revascularization or target vessel myocardial infarction during the 30-day follow-up.

IVL is a potential bail-out treatment in nonexpanded stents with good safety and efficiency profile. On present review of data, IVL is an effective and safe method for facilitating stent expansion and increasing luminal gain.

## Data Availability

The data used to support the findings of this study are restricted by Access Health Care Physicians group in order to protect patient privacy.
